# Reversing functional extinction of mammals prompts a rethink of paradigms about seed fate in arid Australia

**DOI:** 10.1098/rsos.171977

**Published:** 2018-01-31

**Authors:** Charlotte H. Mills, Mike Letnic

**Affiliations:** Centre for Ecosystem Science, School of Biological, Earth and Environmental Sciences, UNSW Australia, Sydney 2052, Australia

**Keywords:** *Acacia ligulata*, *Bettongia penicillata*, *Dodonaea viscosa*, granivory, myrmecochory, seed predation

## Abstract

Functional extinction of once abundant species has frequently preceded understanding of their ecological roles. Consequently, our understanding of ecosystems is prone to shifting baselines because it often relies on observations made on depauperate species assemblages. In Australian deserts, current paradigms are that ants are the dominant granivores, mammals are unimportant seed predators and that myrmecochory in many Australian shrubs is an adaptation to increase dispersal distance and direct seeds to favourable germination sites. Here, we ask whether these paradigms could be artefacts of mammal extinction. We take advantage of a predator-proof reserve within which locally extinct native mammals have been reintroduced to compare seed removal by ants and mammals. Using foraging trays that selectively excluded mammals and ants we show that a reintroduced mammal, the woylie (*Bettongia penicillata*) was at least as important as ants in the removal of seeds of two shrub species (*Dodonaea viscosa* and *Acacia ligulata*). Our results provide evidence that the dominance of ants as granivores and current understanding of the adaptive benefit of myrmecochory in arid Australia may be artefacts of the functional extinction of mammals. Our study shows how reversing functional extinction can provide the opportunity to rethink contemporary understanding of ecological processes.

## 1. Introduction

Population declines of historically abundant species have often preceded understanding of their ecological roles [1]. Consequently, changes in ecosystem processes resulting from the loss of species that are now rare or extinct may go unappreciated [2]. In Australia, around 30 endemic marsupial and rodent species have become extinct in the last 200 years. Many more have undergone range declines and become rare owing primarily to predation by introduced predators [3]. For many of these mammals, it has not been possible to identify shifts in ecosystem processes triggered by their functional extinction. This is because such effects can be difficult, if not impossible, to isolate if there is no opportunity to contrast comparable ecosystems where now rare or extinct mammals are present or absent.

In many of the world's deserts, mammals are key predators and dispersers of seeds [4] and therefore important drivers of vegetation dynamics [5]. By contrast, the paradigm in Australian deserts is that ants are the dominant dispersers and predators of seeds and that mammals are unimportant predators and dispersers of seeds [4,6]. In addition, many Australian arid zone shrubs have well-recognized mutualisms with ants as seed dispersers [7]. Hypothesized adaptive benefits of myrmecochory for arid Australian shrubs are that ants increase dispersal distance and direct seeds to sites favourable for germination, but unlike other continents myrmecochory is thought to accrue little benefit as an adaptation to avoid seed predation [7–9]. However, as noted by Morton [6], most studies examining the primacy of ants and mammals as seed predators and adaptive benefits of myrmecochory in Australian deserts were undertaken in ecosystems in which the native mammal communities had been greatly diminished [3,6].

Rewilding efforts in arid Australia have focused on re-establishing populations of medium-sized marsupials within predator-proof fenced reserves and provide a rare opportunity to study mammal assemblages as they may have existed 200 years ago [10]. In a previous study [11], we found that native mammals within predator-proof exclosures at Arid Recovery (the burrowing bettong, *Bettongia lesueur* and spinifex hopping mouse, *Notomys alexis*) and Scotia Sanctuary (the burrowing bettong, *B. lesueur*) were more significant predators of shrub seeds than ants and that their loss is a likely driver of vegetation changes such as shrub encroachment. In this study, we further the idea that rewilded mammals are significant seed predators by conducting an experiment in a different exclosure to that investigated by Mills *et al.* [11] at Scotia Sanctuary and examining the role of another rewilded mammal, the brush-tailed bettong (*Bettongia pencillata*) as a seed predator. Specifically, we ask if the paradigm that mammals are unimportant seed predators in Australian deserts is an artefact of their historical decline and hence functional extinction. To determine if ants or reintroduced mammals were the dominant seed predators we conducted a foraging tray experiment with treatments that selectively excluded ants or mammals.

## 2. Material and methods

### 2.1. Study site

This research was conducted at Scotia Wildlife Sanctuary (Scotia; −33.20°S, 141.16°E), a conservation reserve run by the Australian Wildlife Conservancy in southwest New South Wales, Australia. Scotia is semi-arid with hot summers and cool winters (mean annual rainfall 286 mm; Australian Bureau of Meteorology 2016).

Scotia boasts two independent 40 km^2^ predator-proof exclosures into which locally extinct marsupials have been reintroduced (stage 1 and stage 2). The bridled nail-tailed wallaby (*Onychogalea fraenata*, body mass 3–6 kg); numbat (*Myrmecobius fasciatus*, 0.3–0.7 kg) and greater bilby (*Macrotis lagotis*, 0.8–2.4 kg) are present in both stage 1 and stage 2. Of the two species of bettong reintroduced at Scotia, the burrowing bettong (*B. lesueur*, 0.9–1.6 kg) is only present in stage 1 while the woylie is only present in stage 2 (*B. penicillata ogilbyi*, 1–1.6 kg). We conducted our experiment within stage 2 of Scotia. Mammal populations in the two exclosures are separated by a fence which they cannot traverse and thus the populations in the two exclosures are independent.

### 2.2. Seed removal experiment

To compare seed removal rates by ants and mammals, we conducted foraging tray experiments in August 2015 (winter) and March 2016 (summer) using seeds of two shrub species that occur locally: *Acacia ligulata* and *Dodonaea viscosa* subsp. *angustissima.* The seed of *A. ligulata* (seed weight: 19 mg) has a large eliaosome which attracts ant and bird dispersers [7]. The seed of *D. viscosa* (seed weight: 10 mg) has a small aril, no eliaosome and is dispersed by ants [8]. Seeds were sourced from a commercial supplier.

At sites spaced 1 km apart we placed five foraging trays along a transect at 20 m intervals. Each foraging tray consisted of a plastic tray (20 cm diameter) buried flush with the ground and filled with sifted soil. Trays were designed to mimic natural deposits of seed that occur under shrubs during fruiting. In summer, we deployed 16 sites for each seed species and in winter we deployed 15 sites for *D. viscosa* and 10 sites for *A. ligulata*.

Foraging trays at each site were randomly assigned one of five treatments: mammal exclusion (caged exclosure), ant exclusion (ring of Coopex® insecticide powder), ant exclusion procedural control (ring of bicarb soda, allowing full access for all taxa), mammal exclusion procedural control (cage with no sides, allowing full access for all taxa) and control (no cage or powder, full access) [12]. The mammal exclusion treatment also excluded birds, however, during a pilot study we found that birds did not visit the foraging trays. In each tray, we placed 50 unblemished seeds of *D. viscosa* or *A. ligulata*. One seed species was used per site. To identify the taxa removing seeds we swept the substrate in a 50 cm circumference around each tray and at collection recorded presence of spoor and if any ants were in the tray. After 48 h, trays were revisited and seeds sifted from sand and counted to determine the number of seeds removed. Visitation was calculated as the percentage of trays at which spoor of taxa (woylie, ant and bird) was detected and the respective taxon had access.

If mammals were significant seed predators and ants insignificant seed predators, we expected to find no difference in seed removal between ant exclusion treatments and control treatments, but lower seed removal from mammal exclusion treatments. If mammals and ants are equally significant seed predators, we expected that seed removal from mammal exclusion treatments and ant exclusion treatments would be equal. If ants were the dominant seed predators and mammals insignificant seed predators we expected no difference in seed removal from mammal exclusion treatments and control treatments, but lower seed removal from ant exclusion treatments. We deployed procedural controls for both exclusion treatments to measure any effects of the exclusion structures on seed removal. If the exclusion structures had no influence on results, we expected to find that seed removal from procedural controls would not differ from controls.

### 2.3. Statistical analysis

To compare the effects of season, treatment and their interaction on seed removal we used a generalized linear mixed-effects model with a Gaussian distribution and site as a random factor. We used Tukey's HSD tests to perform post hoc pairwise comparisons. Analyses were conducted in R v. 3.3.2 [13] using lme4 v. 1.1.12.

## 3. Results

Woylies were the only mammalian predator of seeds. Woylie spoor was detected at 45% of foraged trays containing *D. viscosa* seeds and 34% of foraged trays containing *A. ligulata* seeds. Ants were detected in 15% of *A. ligulata* foraged trays and 2% of *D. viscosa* foraged trays. Bird spoor was not detected at any of the trays.

There was an effect of treatment for both *A. ligulata* (*F*_4, 112_ = 5.66, *p* < 0.001) and *D. viscosa* (*F*_4, 143_ = 10.05, *p* < 0.001). There was an effect of season for both *A. ligulata* (*F*_1, 112_ = 27.09, *p* < 0.001) and *D. viscosa* (*F*_1,143_ = 33.43, *p* < 0.001) and there was an interaction between treatment and season for *A. ligulata* (*F*_1,112_ = 4.81, *p* < 0.001) and for *D. viscosa* (*F*_4, 143_ = 3.76, *p* < 0.01).

**Figure 1.**
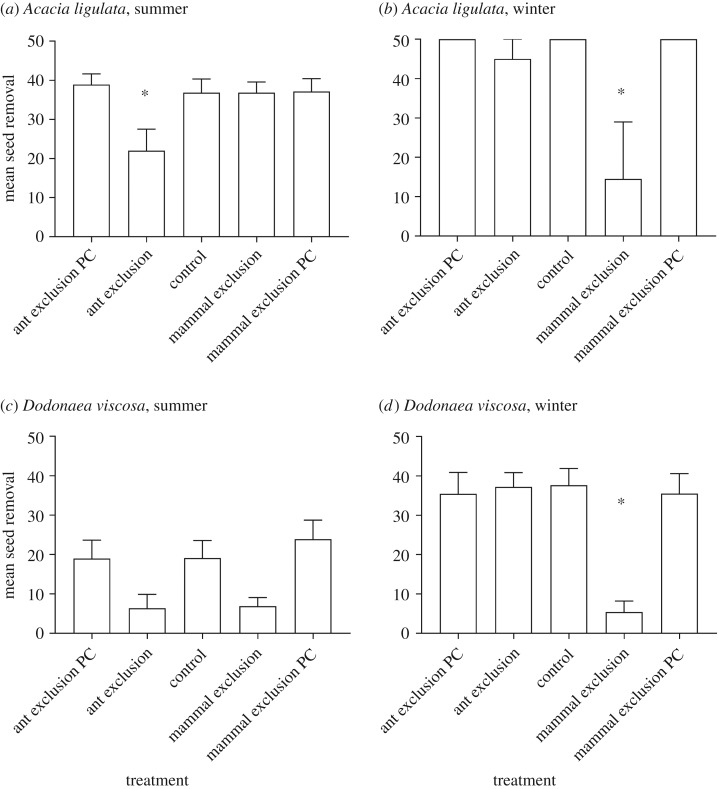
Mean seed removal (+1 s.e.m.) for each treatment for both seed species in both seasons. Total possible seed removal is 50. Asterisks denote significant differences from the Control treatment at *p* < 0.05.

Post hoc pairwise tests revealed that during winter there was less seed removal from mammal exclusion treatments compared to control treatments for *A. ligulata* (*p* < 0.05) (figure 1*b*) and compared to all other treatments for *D. viscosa* (*p* < 0.001) (figure 1*d*), but no difference for other pair combinations (*p* > 0.05). In summer, there was a difference between the ant exclusion and control for *A. ligulata* (*p* < 0.01) (figure 1*a*), but there was no difference between treatments in summer for *D. viscosa* (*p* > 0.05) (figure 1*c*). *Acacia ligulata* seeds had consistently higher removal rates than *D. viscosa*. Overall, seed removal was higher in winter than in summer (figure 1). Procedural controls for both species were not significantly different from controls for summer or winter (*p* > 0.1) (figure 1), indicating that the physical presence of the experimental treatments had no unintended influence on seed removal.

## 4. Discussion

Our results imply that the dominance of ants as granivores in arid Australia [6] may be an artefact of the decline and hence functional extinction of native mammals [3]. The foraging tray experiment revealed that reintroduced woylies were at least as important as ants in the removal of seeds of two shrub species. Moreover, high rates of seed predation from the ant exclusion treatments in winter and for *A. ligulata* in summer suggest that woylies predate on seeds year-round whereas ants primarily removed seeds during summer and took very few seeds during winter. Our experiment provides evidence that woylies are significant seed predators, although it is important to note that the mammal assemblage at Scotia had a depauperate native rodent assemblage with just one species of native rodent present in low density. Because many native rodents are granivorous [11,12,14] it is likely that restoration of native rodent assemblages would increase the overall rate of seed predation by mammals.

Our study highlights how reversing functional extinction can allow us to rethink contemporary ecological processes [15]. In many cases population declines of once abundant species occurred prior to understanding of the roles they fulfilled within ecosystems [1,11,12,16]. Consequently, contemporary understanding of ecosystem processes may be prone to shifting baselines because we simply do not know how species that are now rare or extinct shaped ecosystems in the past [2,11,12].

Taken together with previous studies showing that the burrowing bettong (*B. lesueur*) [11], spinifex hopping mouse (*N. alexis*) [11] and dusky hopping mouse (*Notomys fuscus*) [12] are significant consumers of seeds, our results contribute to a growing body of work demonstrating that where small and medium-sized mammals persist or have been reintroduced they can be significant seed predators. These findings suggest that granivorous mammals may once have been the dominant consumers and removers of seeds across the vast areas of arid Australia where they are now rare or extinct and that their presence or absence may have far-reaching ramifications for seed fate. This is because mammals frequently destroy seeds while consuming them whereas many of the seeds removed by ants are not consumed but simply have their eliaosome removed before they are discarded [7].

Our findings add a new dimension to current thinking about the adaptive benefits that myrmecochory has for arid Australian plants [7–9]. Globally, myrmecochory is thought to provide benefits for plants by dispersing seeds away from sites where they will be vulnerable to predation by granivores, particularly mammals, increasing dispersal distance and directing seeds to microsites suitable for germination [9,17]. However, in arid Australia myrmecochory is thought to accrue little benefit as an adaptation to avoid seed predation [7,8]. This may be because seed predation by mammals had little influence on the fate of seeds in studies that were conducted where granivorous mammals were rare or extinct [6]. By showing that rewilded mammals are significant predators of shrub seeds our study provides support for the idea that myrmecochory in Australian arid zone shrubs may also be an adaptation to escape predation by mammals [9,18].

## Data accessibility

Our data are deposited at Dryad: http://dx.doi.org/10.5061/dryad.gc42g [19].
